# Nicotinamide enhances osteoblast differentiation through activation of the mitochondrial antioxidant defense system

**DOI:** 10.1038/s12276-023-01041-w

**Published:** 2023-07-18

**Authors:** Heein Yoon, Seung Gwa Park, Hyun-Jung Kim, Hye-Rim Shin, Ki-Tae Kim, Young-Dan Cho, Jae-I Moon, Min-Sang Park, Woo-Jin Kim, Hyun-Mo Ryoo

**Affiliations:** 1 Department of Molecular Genetics & Dental Pharmacology, School of Dentistry and Dental Research Institute, Dental Multi-omics Center, Seoul National University, Seoul, 08826 South Korea; 2 Department of Periodontology, School of Dentistry and Dental Research Institute, Seoul National University and Seoul National University Dental Hospital, Seoul, 03080 South Korea

**Keywords:** Energy metabolism, Stress signalling

## Abstract

Although the normal physiological level of oxidative stress is beneficial for maintaining bone homeostasis, imbalance between reactive oxygen species (ROS) production and antioxidant defense can cause various bone diseases. The purpose of this study was to determine whether nicotinamide (NAM), an NAD^+^ precursor, can support the maintenance of bone homeostasis by regulating osteoblasts. Here, we found that NAM enhances osteoblast differentiation and mitochondrial metabolism. NAM increases the expression of antioxidant enzymes, which is due to increased FOXO3A transcriptional activity via SIRT3 activation. NAM has not only a preventive effect against weak and chronic oxidative stress but also a therapeutic effect against strong and acute exposure to H_2_O_2_ in osteoblast differentiation. Collectively, the results indicate that NAM increases mitochondrial biogenesis and antioxidant enzyme expression through activation of the SIRT3-FOXO3A axis, which consequently enhances osteoblast differentiation. These results suggest that NAM could be a potential preventive or therapeutic agent for bone diseases caused by ROS.

## Bone health: Vitamin B3 boosts anti-oxidants

A form of vitamin B3 called nicotinamide (NAM) has potential to prevent or treat bone diseases related to oxidative stress by boosting production of anti-oxidant enzymes. Mitochondria, the powerhouses of the cell, produce reactive oxygen species (ROS) during normal metabolism. If these ROS are not countered by enough anti-oxidants, oxidative stress can decrease production of bone-building cells called osteoblasts, leading to bone aging and osteoporosis. Hyun-Mo Ryoo and Woo-Jin Kim at Seoul National University in South Korea and co-workers investigated whether NAM, the precursor of a key mitochondrial molecule, could restore ROS balance. In experiments in bone cells, NAM improved mitochondrial function, boosted anti-oxidant production, and promoted osteoblast formation. NAM prevented and repaired oxidative damage in bone cells and therefore shows potential both as a preventative and therapeutic treatment for bone health.

## Introduction

Reactive oxygen species (ROS) are highly reactive molecules that are mainly generated from the mitochondrial electron transport chain (ETC)^1^. ROS are inevitably produced during normal metabolic processes as natural byproducts and play various roles in normal physiological processes, activating signaling pathways to initiate biological processes as “secondary messengers” under normal conditions^2^. Imbalance between ROS production and the antioxidant defense system induces oxidative stress^3^.

Maintaining the balance of ROS is also important in bone homeostasis and pathology^4^. SOD2, an antioxidant enzyme, plays a critical role in osteoblast differentiation by regulating mitochondrial ROS at appropriate levels to avoid their accumulation^5^. ROS-induced oxidative stress is also related to bone pathologies, such as bone aging^6^, postmenopausal osteoporosis^7^ and osteoarthritis^8^. Therefore, targeting the dysfunctional mitochondria accompanying ROS imbalance and regulating antioxidant enzymes are potential targets to enhance bone health.

Among the several subcellular organelles that generate ROS, mitochondria are the major sources of intracellular ROS production, accounting for over 90% of ROS^4^. To maintain an appropriate level of ROS, various antioxidant enzymes are involved in scavenging mitochondrial ROS^9^. The highly reactive radical superoxide molecules are catalyzed to a less-radical ROS, hydrogen peroxide, by the activity of the superoxide dismutase (SOD) family. Next, these H_2_O_2_ molecules are further catalyzed into H_2_O by the intervention of antioxidant enzymes such as catalase, thioredoxin peroxidase, and glutathione peroxidase^10^.

Nicotinamide (NAM), the water-soluble form of vitamin B3, has been categorized as a food additive rather than a pharmaceutical and showed a favorable safety profile in a clinical trial^11^. As one of the precursors of nicotinamide adenine dinucleotide (NAD^+^), NAM can increase the level of NAD^+^ just like nicotinic acid (NA), nicotinamide mononucleotide (NMN), and nicotinamide riboside (NR). NAD^+^ is a vital cofactor/coenzyme in regulating cellular metabolism and energy homeostasis, including glycolysis in the cytosol, the tricarboxylic acid (TCA) cycle, oxidative phosphorylation (OXPHOS), and fatty acid and amino acid oxidation in the mitochondria^12^. An increase in NAD^+^ level due to the supplementation of its precursors, such as NAM, NA, and NR, has been reported to induce activation of sirtuins (e.g., SIRT1 and SIRT3) accompanied by activation of transcriptional regulators (e.g., PGC1A and FOXO)^12^ and to increase mitochondrial biogenesis and function^13^. Modulating NAD+ levels using these precursors may protect cells from oxidative stress by enhancing mitochondrial function. In this study, we observed that NAM stimulates osteoblast differentiation, and RNA sequencing (RNA-seq) was performed to fully understand the mechanism underlying NAM-induced osteogenic differentiation. We demonstrate that NAM significantly enhances osteoblast differentiation by alleviating mitochondrial oxidative stress and prevents ROS-induced osteoblast dysfunction.

## Materials and methods

### Cell culture and osteoblast differentiation

MC3T3-E1 cells were cultivated in growth medium composed of α-MEM with 10% fetal bovine serum containing 100 U/mL penicillin and 100 μg/mL streptomycin. After 2 days of cell plating, NAMs were treated with osteogenic media supplemented with 10 mM β-glycerophosphate and 50 μg/mL ascorbic acid in growth media. When inducing oxidative stress, 100 μM H_2_O_2_ was added during NAM treatment. The medium containing NAM and H_2_O_2_ was replaced every other day in every experiment.

### Reagents

A 30% hydrogen peroxide solution (H1009) with nicotinamide (N0636) was purchased from Sigma‒Aldrich (St. Louis, MO, USA).

### Alkaline phosphatase staining and alizarin red S staining

Alkaline phosphatase (ALP) staining and alizarin red S (ARS) staining were performed as described previously^14^. Briefly, an ALP staining kit (COSMO BIO, Tokyo, Japan) was used, and we performed the staining in accordance with the manufacturer’s protocol. For ARS staining, mineralized cells were fixed with 4% paraformaldehyde and stained with 0.5% alizarin red S staining solution, pH 4.2, for 10 min at room temperature.

### Reverse transcription and quantitative real-time polymerase chain reaction

Total RNA extraction from cells, reverse transcription and quantitative real-time polymerase chain reaction were performed as described previously^15^. Total RNA isolation was performed using an RNeasy Mini Kit (Qiagen, Mannheim, Germany). The total RNA was reverse-transcribed into cDNA via PrimeScript^TM^ RT Master Mix (Takara Bio, Shiga, Japan). Takara SYBR Premix Ex Taq (Takara Bio) was used for qPCR in an Applied Biosystems 7500 RT‒PCR system or a StepOnePlus^TM^ Real-Time PCR System. The relative gene expression was determined using standard 2^(-ΔΔCt)^ calculations by normalization to GAPDH. The primers are listed in Supplementary Table 1.

### Subcellular fractionation

NE-PER^TM^ nuclear and cytoplasmic extraction reagents (Life Technologies, CA, USA) were used for nuclear–cytoplasmic fractionation in accordance with the manufacturer’s protocol. The mitochondrial fraction was isolated with a Mitochondria/Cytosol Fractionation kit (Abcam, Cambridge, UK) in accordance with the manufacturer’s protocol.

### siRNA transfection

When MC3T3-E1 cells reached approximately 70–80% confluence, they were transfected with 100 nM siRNA using Lipofectamine RNAiMax Reagent (Invitrogen, USA) in accordance with the manufacturer’s instructions^14^.

### Immunoprecipitation

Cell lysates were precleared by incubation with Pierce^TM^ Protein G Magnetic Beads (Thermo Scientific^TM^, USA) for 30 min on a rotator at 4 °C. The supernatant was incubated overnight with anti-acetyl lysine antibody (Cell Signaling Technology, USA) on a rotator at 4 °C. Protein G magnetic beads were added to the supernatant and incubated for 2 h at 4 °C. These beads were then collected and washed five times with lysis buffer, after which protein sample buffer was added. The samples were subsequently boiled for 10 min and used for immunoblotting^16^.

### Immunoblotting

Cellular proteins obtained using a PRO-PREP protein extraction solution (iNtRON, South Korea) were used for immunoblotting (IB). The proteins were resolved by sodium dodecyl sulfate‒polyacrylamide gel electrophoresis (SDS‒PAGE) and transferred to a polyvinylidene fluoride (PVDF) membrane. Then, 5% nonfat skim milk in PBS containing 0.05% Tween-20 was used for blocking. The membrane was incubated with primary and secondary antibodies and developed via the enhanced chemiluminescence method (Clarity^TM^ Western ECL substrate; Bio-Rad, CA, USA). FUSION FX (VILBER, France) was used for visualization. IB was conducted using antibodies against cytochrome c, TUBA1A (Santa Cruz, Biotechnology, Inc., USA), PGC1A, UCP2, and TRX2 (Proteintech, USA); a Total OXPHOS Rodent WB Antibody Cocktail (Abcam, UK); and antibodies against SOD2, FOXO3A and phospho-FOXO3A (Ser253) (Cell Signaling Technology, USA).

### Oxygen consumption rate assay

MC3T3-E1 cells were plated in XF 96-well microplates (Agilent Technologies, USA). Oxygen consumption was measured with an XF96 Extracellular Flux Analyzer (Agilent Technologies, USA) and Seahorse XF Cell Mito Stress Test Kit. The oxygen consumption rate was measured using 1.5 μM oligomycin, 0.5 μM FCCP, and 0.5 μM rotenone/antimycin A. The results were normalized by counting the number of nuclei through Hoechst 33342 staining (Sigma‒Aldrich, USA).

### Biochemical analysis

The activity of SOD2 was measured using a Superoxide Dismutase Activity Assay kit (Abcam, UK) in accordance with the manufacturer’s protocol. The cellular ATP level was determined by using an ATLP bioluminescence assay kit (Sigma‒Aldrich, USA) in accordance with the manufacturer’s instructions.

### Intracellular ROS determination

Cellular ROS were visualized using CellROX^TM^ Deep Red Reagent (Invitrogen, USA) and observed by confocal microscopy (Zeiss, Germany). MitoSOX^TM^ Red Reagent was used to determine the mitochondrial ROS level. Briefly, MC3T3-E1 cells were seeded on coverslips and cultured with 5 μM NAM in osteogenic medium, and the cultured cells were then stained with 5 μM CellROX^TM^ or MitoSOX^TM^ diluted in HBSS for 20 min at 37 °C. Hoechst 33342 was used to visualize the nucleus. The coverslips were washed three times with HBSS and observed by confocal microscopy (LSM 800 Airyscan; Zeiss, Germany).

### Luciferase reporter assay

The transcription-activating activity of FOXO3A was evaluated with the FHRE-Luc reporter gene purchased from Addgene. Passive lysis buffer, a Bright-Glo Luciferase Assay System, and a GloMax-Multi Detection System (Promega, USA) were used to measure luciferase activity^17^.

### Immunofluorescence staining and confocal microscopy

Immunofluorescence analysis was performed as described previously^14^. In brief, cells were plated and cultured on coverslips. The cells were fixed, permeabilized and stained with the designated primary and secondary antibodies. After 4′,6-diamidino-2-phenylindole (DAPI) staining (Sigma‒Aldrich, USA), the coverslips were mounted on glass slides for visualization using an LSM 800 confocal microscope (Zeiss, Germany)^17^.

### Quantification of γH2AX foci

Quantification of γ-H2AX foci was performed as previously described^14^. Briefly, cells were treated with 10 μM NAM in osteogenic medium and stained with an anti-γH2AX antibody (Cell Signaling Technology, USA). The nuclei were stained with DAPI and visualized via confocal microscopy. The number and intensity of γ-H2AX foci were analyzed by ImageJ software. Cells with 10 or more foci with an intensity of ≥10,000 were counted.

### Flow cytometry

Apoptosis was evaluated using an FITC Annexin V/Dead Cell Apoptosis Kit (Invitrogen, USA) in accordance with the manufacturer’s instructions. MC3T3-E1 cells were treated with H_2_O_2_ and NAM and then harvested and washed with ice-cold PBS. The cells were stained with FITC-labeled annexin V and PI at RT for 10 min and analyzed by a fluorescence-activated cell sorter flow cytometer (FACSverse; BD, USA). At least 20,000 cells were used for each experiment, which was performed in triplicate.

### RNA sequencing analysis

MC3T3-E1 cells were treated with 10 μM NAM in the presence or absence of 100 μM H_2_O_2_ for 4 or 10 days. NAM and H_2_O_2_ were replenished every two days. Total RNA isolation was performed using an RNeasy Mini Kit. The total RNA concentration was calculated using Quant-IT RiboGreen (#R11490; Invitrogen). To assess the integrity of the total RNA, samples were run on TapeStation RNA ScreenTape (#5067-5576; Agilent). High-quality RNAs with an RNA integrity number (RIN) greater than 7.0 were used for RNA library construction.

A library was independently prepared with 1 μg of total RNA for each sample using an Illumina TruSeq Stranded mRNA Sample Prep Kit (#RS-122-2101; Illumina, Inc., San Diego, CA, USA). The first step in the workflow involved purifying the poly‐A-containing mRNA molecules using poly‐T‐attached magnetic beads. Following purification, the mRNA was fragmented into small pieces using divalent cations under an elevated temperature. The cleaved RNA fragments were copied into first strand cDNA using SuperScript II reverse transcriptase (#18064014; Invitrogen) and random primers. This step was followed by second-strand cDNA synthesis using DNA Polymerase I, RNase H and dUTP. These cDNA fragments then underwent an end repair process, the addition of a single ‘A’ base, and ligation of the adapters. The products were then purified and enriched by PCR to create the final cDNA libraries.

The libraries were quantified using KAPA Library Quantification kits for Illumina Sequencing platforms in accordance with the qPCR Quantification Protocol Guide (#KK4854; KAPA BIOSYSTEMS) and qualified using a TapeStation D1000 ScreenTape (# 5067-5582; Agilent Technologies). The indexed libraries were then submitted to Illumina NovaSeq (Illumina, Inc., San Diego, CA, USA), and paired-end (2 × 100 bp) sequencing was performed by Macrogen, Inc. (Seoul, South Korea).

### RNA-sequencing data processing

Paired-end RNA-seq data obtained from three biological replicates were analyzed under the conditions of vehicle (Veh), 10 μM NAM, 100 μM H_2_O_2_, and 100 μM H_2_O_2_ in combination with 10 μM NAM for 4 or 10 days with osteogenic medium in MC3T3-E1 preosteoblasts. The average read depth was 1.2 × 10^7^ read pairs/sample. The reads were aligned to the mouse genome (mm10) using bcbio-nextgen (v1.2.9), which includes Bowtie2 (v2.2.5)^18^, Samtools (v1.13)^19^, and Sambamba (v0.8.2)^20,21^. Expression calling was performed using Salmon (v1.7.0)^22^ and Kallisto (v0.46.2)^23^.

### Differentially expressed gene (DEG), Gene Ontology (GO) and correlation analyses

DESeq2 (v1.32.0)^24^ was used for DEG analysis with an adjusted *P* value cutoff of <0.05 and a fold change cutoff of >1.2. EnrichGO and gofilter of clusterProfiler (v4.0.5)^25^ were used for GO analysis. Correlation analysis was performed to determine the relationship among DEGs included in GO analysis. This analysis used the baseMean, log2-fold change, and statistical parameter information calculated by performing DEG analysis through corrplot (v0.92)^26^.

### Statistics

Each experiment was performed at least two or three times, and representative results are shown in the figures. The significance of differences was evaluated by Student’s *t* test with Prism 9 software (GraphPad Software, USA). The data are presented as the mean ± SD, and differences were considered significant at *P* < 0.05. The *P* values are as follows: *P < 0.05; **P < 0.01; ***P < 0.001; ****P < 0.0001.

## Results

### NAM stimulates osteoblast differentiation

**Fig. 1:**
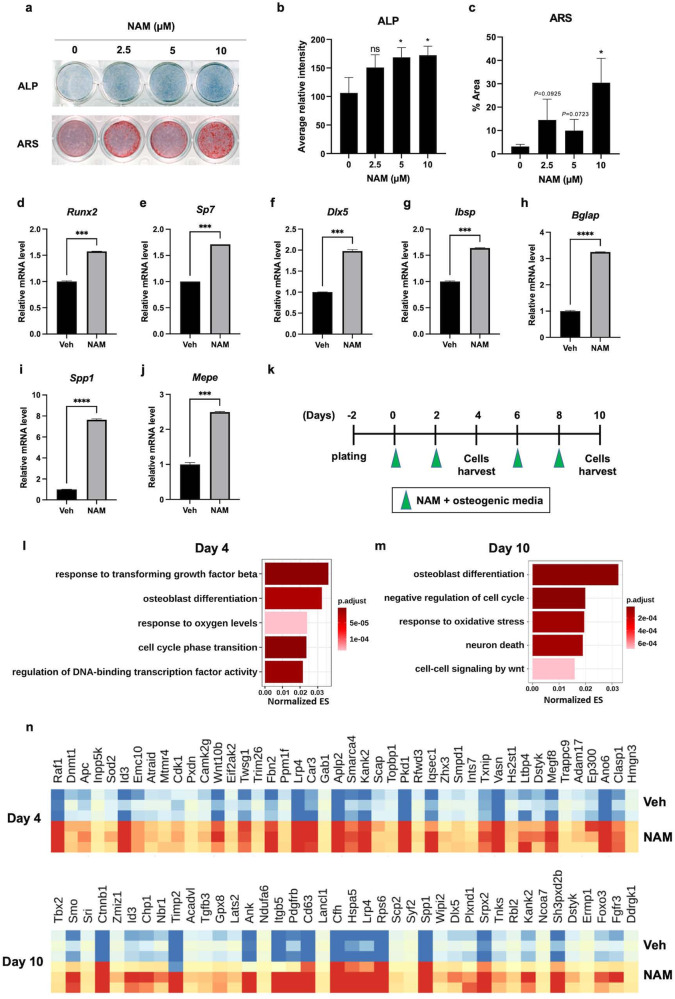
Nicotinamide (NAM) stimulated osteoblast differentiation. **a** ALP and Alizarin Red S (ARS) staining were performed in MC3T3-E1 cells cultured in osteogenic medium supplemented with the indicated concentrations of NAM for 5 and 12 days. The osteogenic medium containing NAM was replaced every other day. **b**, **c** Quantification of each staining was performed by ImageJ. **d**–**j** The mRNA levels of osteoblast differentiation marker genes were determined by RT‒qPCR on Day 10. **k** Scheme of NAM treatment for RNA-seq analysis. MC3T3-E1 cells were cultured with 10 μM NAM in osteogenic medium. The osteogenic medium containing NAM was replaced every two days until the cells were harvested. The green arrows indicate the days of NAM treatment. **l**, **m** Correlation analysis was conducted with NAM-increased DEGs on Days 4 and 10. A normalized correlation matrix is used to show the correlations among GO terms in the biological process category. The GO analysis of genes included in Cluster 4 on Day 4 and Day 10 (yellow box in Supplementary Figs. 2a and 3a) is shown with a bar plot. The top 5 GOs were selected based on the adjusted p values and sorted by enrichment scores. **n** Heatmap of the genes included in Cluster 4 on Day 4 and Day 10 (Supplementary Fig. 2a and 3a). Z score normalization was performed based on statistics calculated by DEseq2. The data are expressed as the mean ± SD. *P < 0.05. **P < 0.01. ***P < 0.001. ****P < 0.0001. ns, not significant.

To investigate whether NAM promotes osteoblast differentiation, MC3T3-E1 osteoblasts were treated with NAM in osteogenic medium. As shown in Fig. 1a, ALP and ARS staining showed that ALP activity and mineralization were enhanced in an NAM dose-dependent manner, and 10 μM NAM had the greatest effect on osteoblast differentiation (Fig. 1b, c). The expression levels of marker genes of osteoblast differentiation, including *Runx2, Sp7, Dlx5*, *Ibsp, Mepe*, *Spp1* and *Bglap*, were significantly increased by NAM treatment (Fig. 1d–j). In particular, the late osteoblast differentiation markers *Bglap*, *Spp1*, and *Mepe* were very highly stimulated by NAM treatment. To understand the mechanism by which NAM stimulated osteoblast differentiation, MC3T3-E1 cells were treated with or without 10 μM NAM during osteogenic differentiation for 4 or 10 days and then subjected to RNA sequencing (RNA-seq) analysis (Fig. 1k). In total, the number of differentially expressed genes (DEGs) was higher on Day 10 (3,858 genes) than on Day 4 (2246 genes). MA plots showed differential gene expression, and the log2(fold change) values of the genes congregated near 0.5 (~1.4 times) on both days (Supplementary Fig. 1a, b). Consistent with the results shown in Fig. 1a, Gene Ontology (GO) analysis of the DEGs upregulated by NAM treatment showed enrichment of GO terms related to osteoblast differentiation and regulation of ossification on both Day 4 and Day 10 (Supplementary Fig. 1c). Given that gene expression correlation analysis can be used to identify functional correlations between genes^27,28^, correlation analysis of DEGs upregulated by NAM was performed here to identify genes associated with NAM-induced osteoblast differentiation (Supplementary Figs. 2a and 3a). On Day 4, osteoblast differentiation and responses to oxygen levels showed correlations in Cluster 4 (Fig. 1l and Supplementary Fig. 2h). Furthermore, genes related to the cell cycle were highly enriched in all clusters (Supplementary Fig. 2b–g). On Day 10, oxidative stress-related genes were included in all clusters (Supplementary Fig. 3b–i), and we found that osteoblast differentiation and the response to oxidative stress were associated in Cluster 4 (Fig. 1m and Supplementary Fig. 3j). The genes affiliated with Cluster 4 are shown using a heatmap (Fig. 1n). These genes included *Ndufa6*, *Gpx8*, and *Foxo3a*, which are involved in regulating oxidative stress, as well as *Sod2*, which encodes a mitochondrial antioxidant enzyme. These results suggest that the enhancement of osteoblast differentiation by NAM might be related to regulation of oxidative stress.

### NAM relieves mitochondrial ROS by enhancing the expression or activity mitochondrial antioxidant enzymes

**Fig. 2:**
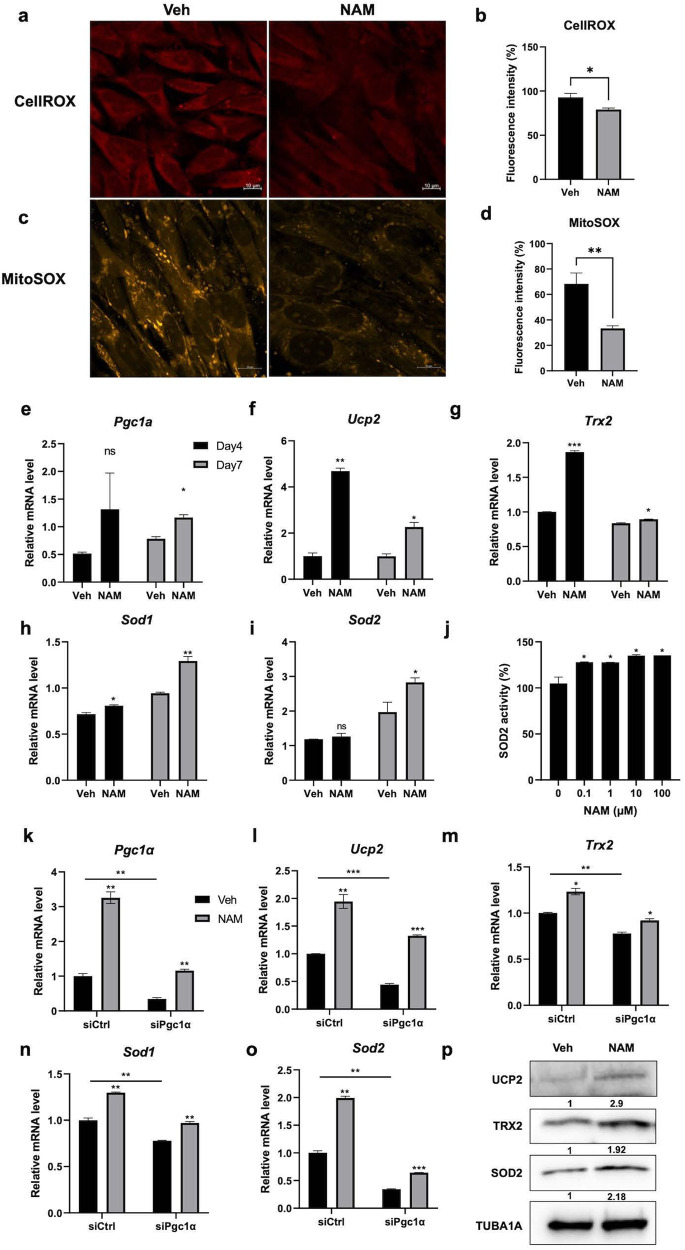
NAM reduces mitochondrial ROS levels by increasing antioxidant enzymes. **a** The oxidative stress in MC3T3-E1 cells was determined by CellROX^TM^ reagent after 10 μM NAM treatment with osteogenic media for 7 d. **b** The fluorescence intensity of (**a**) was calculated via ImageJ. **c** Mitochondrial superoxide was detected by MitoSOX^TM^ mitochondrial superoxide indicator in MC3T3-E1 cells after 10 μM NAM treatment in osteogenic media for 7 d. **d** The fluorescence intensity of (**c**) was measured by ImageJ. **e**–**i** The mRNA expression levels of Pgc1α and ROS scavenger enzymes in MC3T3-E1 cells cultured in osteogenic medium supplemented with or without 10 μM NAM for 4 and 7 d were determined by RT‒qPCR. **j** The mitochondrial fraction was isolated from the culture under the same conditions to measure SOD2 enzymatic activity. **k**–**o** To understand the influence of PGC1A on ROS scavenger enzyme gene expression, MC3T3-E1 cells transfected with siCtrl or siPgc1α were cultured in osteogenic medium with or without 10 μM NAM for 4 d. **p** MC3T3-E1 cells were cultured in osteogenic medium with or without 10 μM NAM for 7 d. The protein expression level of ROS scavenger enzymes was determined by immunoblot analysis. Data are expressed as mean ± SD. *P < 0.05. **P < 0.01. ***P < 0.001. ****P < 0.0001. ns, not significant.

To determine the effect of NAM on the cellular oxidative stress level, MC3T3-E1 cells were stained with a fluorogenic probe, CellROX^TM^, which measures the ROS level in the cytosol, and MitoSOX^TM^, which measures superoxide production in mitochondria. NAM treatment significantly reduced ROS accumulation in the cytosol (Fig. 2a, b) and superoxide production in the mitochondria (Fig. 2c, d).

PGC1A is a transcriptional coactivator that plays a central role in the expression of genes acting against oxidative stress in combination with FOXO3A^29^. PGC1A is also known to promote mitochondrial biogenesis and enhance the capacity of cells to detoxify ROS, allowing cells to efficiently produce ATP and respire with less oxidative stress^30^. For this reason, we measured the mRNA levels of *Pgc1α* and its downstream detoxifying genes in MC3T3-E1 cells treated with NAM for 4 and 7 days. NAM increased the expression levels of *Pgc1α*, *Ucp2*, *Trx2*, *Sod1* and *Sod2* in differentiating MC3T3-E1 cells (Fig. 2e–i). NAM also significantly increased the enzymatic activity of SOD2, a key mitochondrial antioxidant enzyme, in differentiating MC3T3-E1 osteoblast cells (Fig. 2j).

To investigate whether NAM upregulates the expression of ROS-detoxifying enzymes through PGC1A, MC3T3-E1 cells were treated with small interfering RNAs (siRNAs) targeting *Pgc1α*. siPgc1α efficiently knocked down *Pgc1-α* expression, and NAM increased *Pgc1α* expression in both siCtrl- and siPgc1α-transfected MC3T3-E1 cells (Fig. 2k). As previously reported, *Pgc1-α* knockdown significantly decreased the mRNA levels of *Ucp2*, *Trx2*, *Sod1* and *Sod2* (Fig. 2l–o). The increase in the expression of mRNAs encoding ROS-detoxifying enzymes by NAM in siPgc1α-transfected cells was much lower than that in siCtrl-transfected cells. Consistent with this, the protein levels of ROS-detoxifying enzymes were increased by NAM treatment in MC3T3-E1 osteoblast cells (Fig. 2p). These results suggest that NAM relieves mitochondrial oxidative stress by inducing the expression of antioxidant enzymes.

### NAM induces antioxidant enzyme expression through the SIRT3/FOXO3A axis

**Fig. 3:**
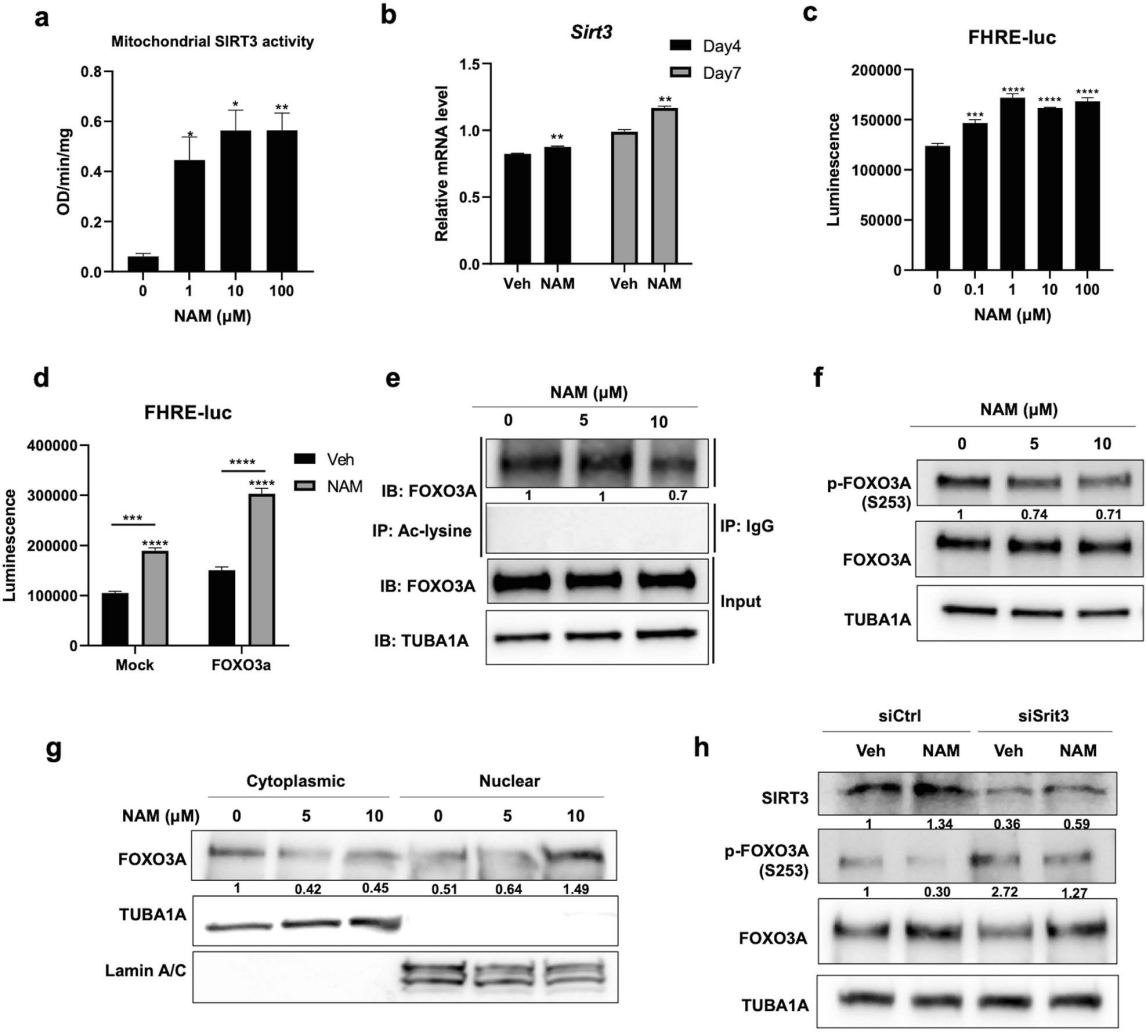
NAM-induced SIRT3 and FOXO3A activation mediates the upregulation of mitochondrial antioxidants. **a** The mitochondrial fraction was isolated after 10 μM NAM treatment with osteogenic medium for 1 d. SIRT3 activity was determined with the mitochondrial fraction via a SIRT activity assay kit. **b** MC3T3-E1 cells were treated with 10 μM NAM in osteogenic medium for 4 and 7 d. The mRNA level of *Sirt3* was determined by RT‒qPCR. **c** The transactivation activity of FOXO3A was measured in MC3T3-E1 cells transfected with FHRE-Luc after NAM treatment for 2 d. **d** Luciferase activity was determined after co-transfection of an FHRE-Luc plasmid and a control plasmid (pcDNA3.1) or with plasmids expressing FOXO3A and treatment with NAM for 2 d. **e** The acetylation level of FOXO3A was determined by immunoprecipitation (IP) with an anti-acetylated-lysine antibody followed by immunoblot analysis with an anti-FOXO3A antibody. **f** MC3T3-E1 cells were treated with the indicated concentrations of NAM for 4 d in osteogenic medium, and cell lysates were immunoblotted with p-FOXO3A (S253) and FOXO3A antibodies. **g** MC3T3-E1 cells were treated with NAM in osteogenic medium for 4 d, after which they were subjected to subcellular fractionation. The subcellular localization of FOXO3A was determined by immunoblot analysis. **h** MC3T3-E1 cells were transfected with siCtrl or siSirt3. The cells were cultured in osteogenic medium with or without 10 μM NAM for 4 d, and the cell lysates were immunoblotted with SIRT3, p-FOXO3A (S253) and FOXO3A antibodies. The data are expressed as the mean ± SD. *P < 0.05. **P < 0.01. ***P < 0.001. ****P < 0.0001. ns, not significant.

Treatment with nicotinamide riboside (NR), an NAD^+^ precursor, is known to increase NAD^+^ levels and activate SIRT3, an NAD^+^-dependent protein deacetylase^31^. NAM has also been found to activate SIRT1, resulting in improved liver function^32^. Because SIRT3 is located in the mitochondria and regulates mitochondrial functions^33^, we tested whether NAM treatment also promotes SIRT3 activity in osteoblast cells. MC3T3-E1 osteoblast cells were treated with NAM, and the mitochondrial fraction was isolated to determine mitochondrial SIRT3 activity. The results showed that such activity was increased by NAM treatment in a dose-dependent manner in MC3T3-E1 cells (Fig. 3a). The mRNA expression of *Sirt3* was also significantly upregulated by treatment with 10 μM NAM in MC3T3-E1 cells (Fig. 3b). SIRT3 promotes the transcription-activating activity of FOXO3A by decreasing the latter’s acetylation and phosphorylation, which in turn induces the expression of genes encoding antioxidant enzymes^34^. We investigated whether NAM regulates the activity of FOXO3A by using the FOXO3A reporter plasmid FHRE-Luc. NAM significantly increased the transcription-activating activity of both endogenous FOXO3a (Fig. 3c) and exogenously transfected FOXO3A in MC3T3-E1 osteoblast cells (Fig. 3d). SIRT3 physically interacts with FOXO3 in mitochondria and promotes the transcriptional activity of FOXO3A^35^. SIRT3 regulates the deacetylation of FOXO3A, which is followed by reduction of its phosphorylation, ubiquitination, and degradation^36^. Moreover, FOXO3A-dependent mitochondrial enzymes, such as SOD2, PRX3, PRX5 and TRX2, are involved in ROS detoxification. The acetylation of FOXO3A was diminished by NAM treatment in MC3T3-E1 cells (Fig. 3e). Dephosphorylation of FOXO3A at S253 has also been reported to stimulate the translocation of FOXO3A from the cytoplasm to the nucleus^37^. The phosphorylation level of FOXO3A (S253) was decreased by NAM in a dose-dependent manner without affecting the FOXO3A protein level (Fig. 3f). Next, we examined whether NAM regulates the subcellular localization of FOXO3A. NAM treatment increased the translocation of FOXO3A from the cytoplasm to the nucleus in MC3T3-E1 cells (Fig. 3g). To investigate whether NAM induces dephosphorylation of FOXO3A (S253) through SIRT3, MC3T3-E1 cells were treated with siRNAs targeting *Sirt3*. While siSirt3 knocked down the expression of SIRT3, NAM slightly increased SIRT3 expression in both siCtrl- and siSirt3-transfected cells (Fig. 3h). As previously reported^36^, *Sirt3* knockdown significantly increased the phosphorylation of FOXO3A at S253. The decrease in the phosphorylation of FOXO3A (S253) by NAM in siSirt3-transfected cells was much lower than that in siCtrl-transfected cells. Taken together, these findings indicate that NAM activates FOXO3A through SIRT3, which in turn promotes the expression of mitochondrial antioxidant enzymes to facilitate ROS detoxification in osteoblasts.

### NAM improves mitochondrial function during osteoblast differentiation

**Fig. 4:**
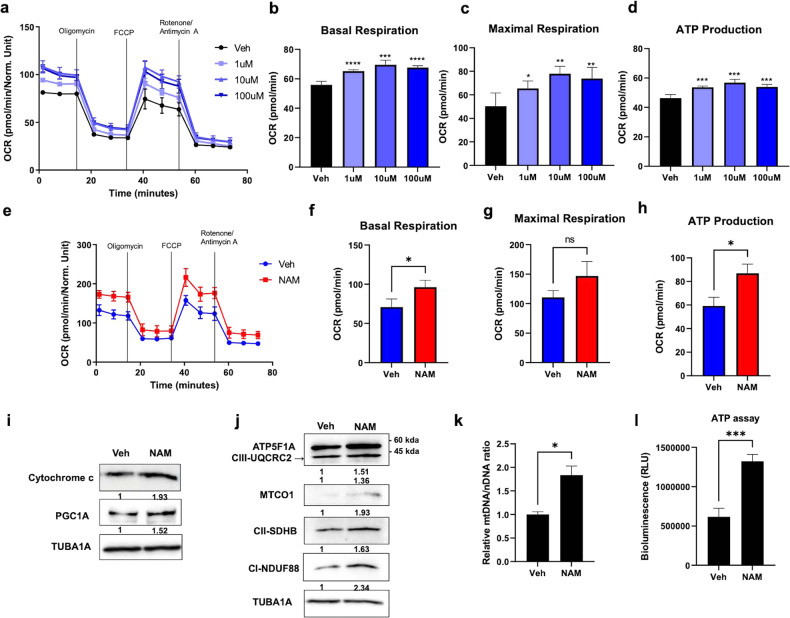
NAM enhances mitochondrial function in osteoblast differentiation. **a**–**d** MC3T3-E1 cells were cultured with the indicated concentrations of NAM in growth medium for 1 d, and the OCR was measured. **e** MC3T3-E1 cells were cultured in osteogenic medium supplemented with or without 10 µM NAM for 7 days, and the OCR was measured and analyzed with bar plots (**f**–**h**). **i** The protein expression levels of Cytochrome C and PGC1A under the same culture conditions were assessed by immunoblot analysis. **j** The protein levels of OXPHOS mitochondrial complexes were measured by immunoblot analysis using a total OXPHOS Rodent WB antibody cocktail. **k** The relative copy numbers of mitochondrial DNA (mtDNA) and nuclear DNA (nDNA) from the same culture condition were evaluated by RT‒qPCR. The mtDNA/nDNA ratio was calculated by comparing the ΔΔCt values. **l** MC3T3-E1 cells cultured under the same conditions were measured for ATP levels. The data are expressed as the mean ± SD. *P < 0.05. **P < 0.01. ***P < 0.001. ****P < 0.0001. ns, not significant.

Excessive oxidative stress reduces mitochondrial function in osteoblasts^38^. Therefore, we tested whether NAM also regulates mitochondrial function in osteoblasts. First, to investigate the effect of osteoblast differentiation on mitochondrial respiration, we measured the oxygen consumption rate (OCR) before differentiation (Day 0) and on Days 4, 7, and 14 of differentiation of MC3T3-E1 cells. The OCR significantly increased during the differentiation period (Supplementary Fig. 4a–h). As NAM improved mitochondrial respiration in undifferentiated osteoblasts (Fig. 4a–d and Supplementary Fig. 4i–l), we investigated whether it can reinforce mitochondrial respiration in differentiating osteoblast cells. NAM was applied to MC3T3-E1 cells during osteogenic differentiation. NAM increased the OCR in differentiating MC3T3-E1 cells cultured in differentiation medium for 7 days (Fig. 4e–h and Supplementary Fig. 4m–p). Because the mitochondrial OCR was increased by NAM treatment, we investigated the changes in the expression of mitochondrial biogenesis-related marker proteins. The protein levels of cytochrome c and peroxisome proliferator-activated receptor γ coactivator-1α (PGC1A) were increased by treatment with 10 μM NAM during the differentiation of MC3T3-E1 cells for 7 days (Fig. 4i). In addition, the levels of other OXPHOS-related proteins were examined using an antibody cocktail that recognizes subunits of each mitochondrial respiratory chain complex: complex I subunit NDUF88, complex II SDHB, complex III UQCRC2, complex IV MTCO1, and ATP5F1A. NAM increased the protein levels of all subunits of complexes I-V compared with those in vehicle-treated cells (Fig. 4j). We further quantified the mtDNA:nDNA ratio after amplification of the mitochondrial and nuclear genomes, as this is another representative marker of mitochondrial biogenesis^39,40^. The results showed that NAM significantly increased this ratio (Fig. 4k). Consistent with this finding, NAM treatment also significantly increased the ATP level in MC3T3-E1 osteoblast cells (Fig. 4l). These results indicate that NAM enhances mitochondrial function, which plays an important role in supplying the energy necessary for osteogenic differentiation.

### NAM prevents osteoblast damage induced by H_2_O_2_ oxidative stress

**Fig. 5:**
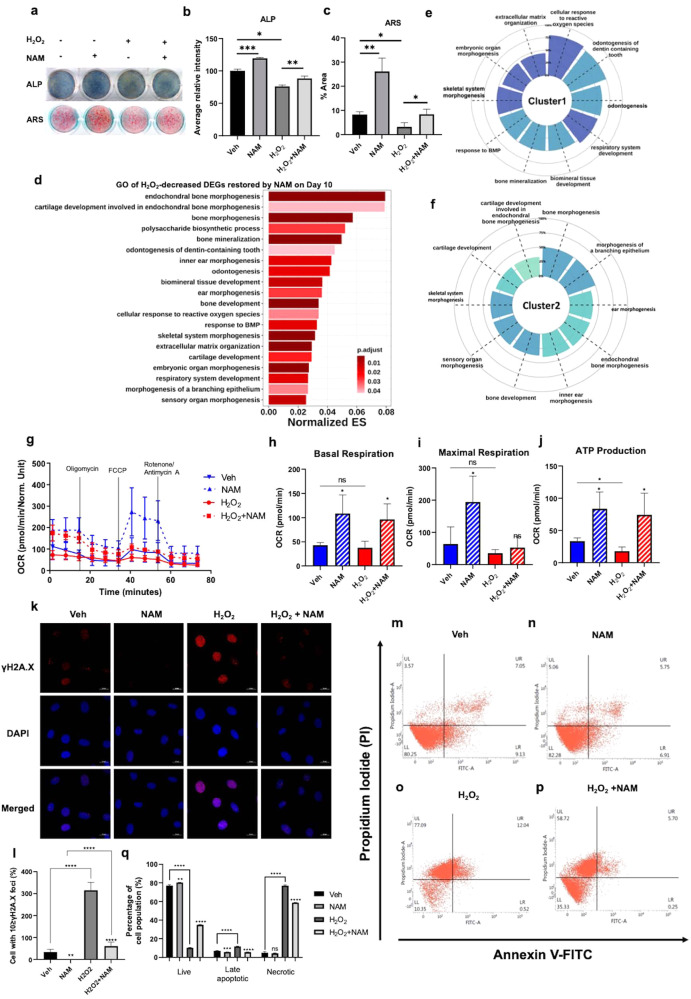
NAM prevents ROS-induced mitochondrial and functional impairment in osteoblasts. **a** ALP staining and ARS staining were performed after 10 μM NAM treatment with osteogenic medium in the presence or absence of 100 μM H_2_O_2_ for 5 and 12 days. **b**, **c** Quantification of each staining was performed by ImageJ. **d** H_2_O_2_-decreased DEGs restored by NAM were investigated by GO analysis in biological process on Day 10. The top 20 GO terms were selected and listed based on the adjusted *P* value and enrichment score. **e**, **f** Correlation analysis was performed on the genes in (**d**). The normalized correlation matrix was created to show the correlations between GO terms. The circular bar plots depict the ratios of genes associated with each GO term. **g** The mitochondrial OCR was evaluated after treatment with 10 μM NAM with or without 100 μM H_2_O_2_ for 7 d in osteogenic medium using an XF96 Extracellular Flux Analyzer. **h**–**j** The parameters calculated from the curved OCR plot are described in bar plots. **k**–**l** MC3T3-E1 cells were treated with 100 μM H_2_O_2_ alone or in combination with 10 μM NAM for 24 h. Immunostaining was performed using a γH2AX antibody (red) and DAPI (blue). To quantify the number of cells with γH2AX foci, the cells containing ≥10 foci were counted. **m**–**p** MC3T3-E1 cells were treated with 300 μM H_2_O_2_ in combination with or without 10 μM NAM. To detect H_2_O_2_-induced cell death, flow cytometry analysis after Annexin V and PI double staining was performed. **q** The percentages of each cell population are presented in a bar plot (*n* = 3). The data are expressed as the mean ± SD. *P < 0.05. **P < 0.01. ***P < 0.001. ****P < 0.0001. ns, not significant.

Accumulation of oxidative stress is strongly related to reduced bone mineral density and impaired osteoblast differentiation^14,41^. Because we observed that NAM induced the expression of mitochondrial antioxidant enzymes in osteoblasts, we tested whether NAM treatment relieves ROS-damaged osteoblasts. MC3T3-E1 cells were treated with NAM in the presence or absence of 100 μM H_2_O_2_ during osteogenic differentiation for 4 or 10 days, and the medium was changed every other day (Supplementary Fig. 5a). ALP and ARS staining results showed that NAM prevented the impairment of osteoblast differentiation caused by chronic exposure to 100 μM H_2_O_2_ (Fig. 5a–c). RNA-seq analysis was performed to identify the effect of NAM on osteoblasts damaged by H_2_O_2_. When the cells were treated with only H_2_O_2_ for 4 days, 405 genes were upregulated, and 228 genes were downregulated (Supplementary Fig. 5b, c). Meanwhile, on Day 10, 247 genes were upregulated and 298 genes were downregulated by H_2_O_2_ treatment (Supplementary Fig. 5d, e). The genes downregulated by H_2_O_2_ were associated with GO terms related to osteoblast differentiation on both Day 4 and Day 10, particularly on Day 10 (Supplementary Fig. 5b, d). To determine whether NAM enables osteoblasts to recover from ROS-induced damage, GO analysis was performed on genes whose expression was decreased by H_2_O_2_ but restored by NAM (Fig. 5d). On Day 4, the expression of only six genes was restored by NAM (Supplementary Fig. 6a). However, on Day 10, the expression of 114 genes was significantly restored by NAM, and these genes were particularly associated with GO terms related to osteoblast differentiation or bone formation (Fig. 5d). A correlation test was performed on the DEGs whose expression was decreased by H_2_O_2_ but restored by NAM, which was included in the top 20 GO terms (Supplementary Fig. 6b). Next, we listed the top 10 ranked GO terms in each cluster (Fig. 5e, f, and Supplementary Fig. 6c). Interestingly, the cellular response to reactive oxygen species and bone mineralization were related in the first cluster (Fig. 5e). Overall, the expression of 80% of genes involved in the cellular response to reactive oxygen species was decreased by H_2_O_2_ but restored by cotreatment with H_2_O_2_ and NAM (Fig. 5e). In addition, Cluster 1 contained *Runx2* and *Wnt10b*, which are crucial factors in osteoblast differentiation (Supplementary Table 2). Cluster 2 included genes associated with many GO categories related to bone development and cartilage development (Fig. 5f), such as *Mmp13*, *Vdr* and *Dlx5* (Supplementary Table 3). Genes in Cluster 3 were related to GO categories, including cartilage development, bone development, and extracellular matrix organization (Supplementary Fig. 6c). Our RNA-seq results showed that the expression of the *Col1A1*, *Col1A2*, and *Vdr* genes associated with osteoporosis (as revealed by OMIM disease enrichment analysis) was decreased by H_2_O_2_ (Table 1). Interestingly, NAM attenuated the H_2_O_2_-induced decreases in the expression of *Col1A1* and *Vdr* (Table 2). These results suggest that NAM can prevent osteoblast dysfunction caused by oxidative stress.

**Table 1:** Enrichment analysis based on OMIM disease libraries for H_2_O_2_-decreased DEGs.

Term	Overlap	Adjusted *P* value	Combined Score	Genes
Osteoporosis	3/11	0.007417265	190.5643617	COL1A1;COL1A2;VDR
Ehlers-Danlos	3/11	0.007417265	190.5643617	COL1A1;COL1A2;TNXB
Hypertension	2/29	0.459410905	13.16195516	PTGIS;AGT
Alopecia	1/11	0.459410905	12.47958022	HR
Hypothyroidism	1/11	0.459410905	12.47958022	TG
Skin/hair/eye pigmentation	1/12	0.459410905	10.86386938	MC1R
Long qt syndrome	1/12	0.459410905	10.86386938	KCNH2
Anomalies	1/13	0.459410905	9.555886574	RUNX2
Rheumatoid arthritis	1/13	0.459410905	9.555886574	PTPN22

**Table 2:** Enrichment analysis based on OMIM disease libraries for H_2_O_2_-decreased DEGs restored by NAM.

Term	Overlap	Adjusted *P* value	Combined Score	Genes
Osteoporosis	2/11	0.017127866	251.2087451	COL1A2;VDR
Hypothyroidism	1/11	0.136997884	49.20758107	TG
Ehlers-Danlos	1/11	0.136997884	49.20758107	COL1A2
Anomalies	1/13	0.136997884	38.63591875	RUNX2
Myopia	1/15	0.136997884	31.38589486	COL2A1
Hypogonadism	1/15	0.136997884	31.38589486	CHD7
Lymphoma	1/22	0.165094954	17.87363593	BCL7A
Deafness	2/111	0.165094954	6.558998122	TMIE;COL2A1
Obesity	1/31	0.1805597	10.64289988	SDC3
Neuropathy	1/35	0.181466527	8.818608039	SLC12A6

ROS are known to impede mitochondrial function^42^. Therefore, we determined whether NAM could regulate ROS-damaged mitochondrial function. NAM prevented the reductions in mitochondrial respiration and oxygen consumption rate caused by treating MC3T3-E1 osteoblast cells with H_2_O_2_ (Fig. 5g–j and Supplementary Fig. 6d–g).

The accumulation of γH2AX foci, which involves histone H2A.X phosphorylation, is a biomarker for DNA damage and genotoxicity accompanying the DNA damage response (DDR)^43^. As previously reported^14^, H_2_O_2_ treatment increased the number of MC3T3-E1 osteoblast cells with γH2AX foci (Fig. 5k–l). NAM significantly ameliorated the accumulation of γH2AX foci that occurred at the basal level as well as that induced by H_2_O_2_ treatment. Excessive ROS can result in apoptosis accompanied by the release of cytochrome c from damaged mitochondria, which in turn disrupts redox homeostasis in tissues^44,45^. We investigated whether NAM alleviates the apoptosis of osteoblasts damaged by acute exposure to excessive H_2_O_2_. Treatment with 300 μM H_2_O_2_ caused acute oxidative damage that significantly decreased the population of Annexin V- and propidium iodide (PI)-negative healthy live cells but significantly increased the numbers of both Annexin V- and PI-positive late apoptotic cells and PI-positive and Annexin V-negative necrotic cells (Fig. 5m–q). Cotreatment with H_2_O_2_ and NAM significantly increased the population of live cells compared with that upon H_2_O_2_ treatment alone. Taken together, these findings indicate that NAM prevents osteoblasts from suffering H_2_O_2_-induced acute or chronic oxidative damage to mitochondria and DNA.

## Discussion

Mitochondria are key intracellular organelles for the generation and regulation of cellular bioenergetics and produce the majority of ATP molecules via the OXPHOS system. Mitochondrial metabolism normally produces ROS as byproducts. Although intracellular ROS are necessary for regular function^46^, when they are present at excess levels, they can lead to intracellular stress and numerous bone disorders, including osteoporosis^47^ and osteoarthritis^48^. To maintain an appropriate level of ROS, antioxidant enzymes play a crucial role in the antioxidant system. A previous study has reported that osteoblast lineage-specific *Sod2*-deficient mice show decreased osteoblast activity accompanied by an osteoporosis-like phenotype^49^. The antioxidant *N*-acetylcysteine, a precursor of glutathione (GSH), also increases osteoblast differentiation in mouse calvarial cells^50^. In this study, we showed that the stimulation of osteoblast differentiation by NAM was related to genes involved in the regulation of oxidative stress by RNA-seq. In addition, via in vitro analysis, we confirmed that NAM alleviated the ROS level in osteoblasts by regulating antioxidant enzymes. Moreover, NAM effectively eliminated ROS that arose under physiological conditions as well as those that emerged acutely through activation of these enzymes. As a result, the induction of antioxidant enzymes by NAM not only strengthened the function of osteoblasts under normal conditions but also significantly prevented the decrease in osteoblast function caused by excessive ROS.

Mitochondria generate ATP through respiration to provide the energy needed for cell metabolism. Therefore, when cells need more energy to function, mitochondrial respiration is stimulated to supply more ATP^51^. Bioenergetic demand and capacity alter as cell function changes^52^. To satisfy this, the mitochondrial capacity and efficiency increase in differentiating cells. The mitochondrial respiration of osteoblasts is elevated during differentiation to provide the required energy. When bone marrow stromal cells (BMSCs) undergo osteogenic differentiation, the mitochondrial OXPHOS reaction is upregulated in BMSCs to fulfill the energetic demands of the process^53,54^. Suppression of mitochondrial respiration using a mitochondrial complex inhibitor (e.g., rotenone) has been reported to inhibit osteoblast differentiation^5^. Our results also showed that mitochondrial respiration was increased during osteoblast differentiation (Fig. 1). Furthermore, NAM increased mitochondrial respiration during osteoblast differentiation (Fig. 2). These results indicate that NAM activates the energy generation required for osteoblast differentiation through increased mitochondrial respiration. The administration of NMN, similar to the exogenous addition of NAD^+^, led to increases in OCR and mitochondrial function in neuroblastoma cells^55^. Thus, NAM has the potential to be used as a medication for the prevention and treatment of bone disorders because it can improve the physiological function of osteoblasts through the metabolic activation of mitochondria.

In bone, osteogenesis is a sequential process of mesenchymal stem cell (MSC) recruitment, preosteoblast proliferation, lineage commitment, collagen secretion, and extracellular matrix mineralization^56^. During this process, the proliferation of MSCs and preosteoblasts is a prerequisite for osteoblast differentiation^57^. Our results of GO analysis of DEGs whose expression was increased by NAM on Day 4, in the early stage of differentiation, revealed that these genes were particularly associated with the cell cycle and chromosome segregation, indicating that NAM might play an important role in ensuring a sufficient population of osteoblast precursors for osteogenesis by regulating genes involved in cell proliferation in the early stage of differentiation. When NAM was supplied in osteogenic medium for 10 days (during the maturation stage of osteoblast differentiation), RNA-seq analysis revealed that along with those related to osteoblast differentiation, a set of genes related to oxidative stress were also significantly affected (Fig. 1). Our data also confirmed that NAM reduced the mitochondrial ROS levels during osteoblast differentiation (Fig. 2) and mitigated mitochondrial and cellular damage caused by excessive ROS (Fig. 5). These results suggest that the supply of NAM is advantageous for improving the functionality of osteoblasts and maintaining bone homeostasis by reducing intracellular ROS levels.

HDACs, including both class I^58^ and class II HDACs^59^, inhibit osteoblast differentiation and bone formation. In addition, MS-275, a class I HDAC inhibitor, has been reported to promote bone formation^60,61^. However, Sirt3, a major deacetylase of mitochondrial Sirts, has been shown to be crucial for bone development and metabolism^62^. Although SIRT3-deficient mice showed bone loss and SIRT3 has been linked to the production of osteoclasts^63^, the function and importance of this protein in osteoblasts are incompletely understood. In this study, we showed that NAM promotes osteoblast differentiation by inducing the expression of antioxidant enzymes through activation of Sirt3 (Fig. 4). In addition, Sirt3, together with FOXO3A, was shown to play an important role in increasing antioxidant enzymes in osteoblasts. FOXO3A induces the expression of antioxidant enzymes under oxidative stress^29^. FOXO3A also inhibits the differentiation of osteoblast precursors by inhibiting Wnt signaling^64^ while inhibiting ROS in mature osteoblasts to reduce apoptosis and promote bone formation^65^. In our study, we showed that NAM promotes osteoblast differentiation by activating FOXO3A and increasing the levels of antioxidant enzymes (Fig. 4). Ultimately, NAM is thought to play an important role in maintaining homeostasis by promoting the expression of antioxidant enzymes in mature osteoblasts. Taken together, the obtained findings suggest that NAM promotes the differentiation of osteoblasts and maintains homeostasis by increasing the levels of antioxidant enzymes through SIRT3 activation and sequential FOXO3A activation.

Oxidative stress is one of the most important factors accelerating aging of the musculoskeletal system^66^. NAD^+^, the levels of which are known to decrease with aging^67^, promotes mitochondrial function and prolongs the lifespan of mice when its levels are increased through NAD precursors such as NR^68^. In this study, we revealed that NAM plays an important role in maintaining mitochondrial homeostasis by regulating antioxidant enzymes to ensure appropriate levels of intracellular ROS. In addition, oxidative stress causes DNA damage and cell death. If DNA requiring repair via the DNA damage response (DDR) continues to accumulate in cells, it cannot be removed through the DNA repair mechanism, leading to cellular senescence via irreversible cell cycle arrest^69^. In our study, NAM prevented osteoblast DNA damage and cell death caused by oxidative stress. Therefore, NAM can prevent the acceleration of musculoskeletal aging caused by oxidative stress.

**Fig. 6:**
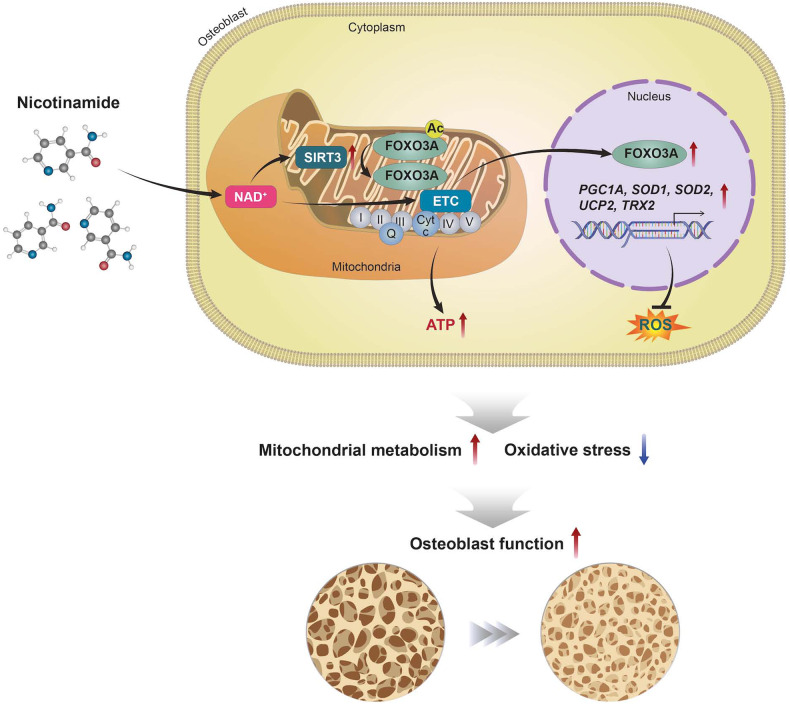
NAM improves osteoblast differentiation and mitochondrial metabolism. NAM increases mitochondrial biogenesis and antioxidant enzyme expression through activation of the SIRT3, FOXO3A, and PGC1A, which consequently enhances osteoblast differentiation. NAM facilitates osteogenic differentiation both in normal physiological conditions and under oxidative stress.

In summary, the present study shows that NAM significantly enhances osteoblast differentiation by relieving mitochondrial oxidative stress (Fig. 6). NAM increases mitochondrial respiration and the expression of antioxidant enzymes via SIRT3, FOXO3A and PGC1A activation, facilitating osteoblast differentiation both in normal physiological conditions and under oxidative stress. On the basis of this study, NAM could be a therapeutic or prophylactic drug to improve bone health.

## Supplementary information

supplemental material
