# Supplementary material for: Nicotinamide enhances osteoblast differentiation through activation of the mitochondrial antioxidant defense system
Source: Exp Mol Med. 2023 Jul 18;55(7):1531–43. doi: 10.1038/s12276-023-01041-w (PMC10393969; doi:10.1038/s12276-023-01041-w)
Supplement: Supplementary file 1 — supplemental material [file 12276_2023_1041_MOESM1_ESM.docx]

**Supplemental information**

**Nicotinamide enhances osteoblast differentiation through activation of the mitochondrial antioxidant defense system**

Heein Yoon^1^, Seung Gwa Park^1^, Hyun-Jung Kim^1^, Hye-Rim Shin^1^, Ki-Tae Kim^1^, Young-Dan Cho^1,2^, Jae-I Moon^1^, Min-Sang Park^1^, Woo-Jin Kim^1,*^, and Hyun-Mo Ryoo^1,*^

^1^ Department of Molecular Genetics & Dental Pharmacology, School of Dentistry and Dental Research Institute, Dental Multi-omics Center, Seoul National University, Seoul (08826), South Korea

^2^ Department of Periodontology, School of Dentistry and Dental Research Institute, Seoul National University and Soul National University Dental Hospital, Seoul (03080), South Korea

*Correspondence: hmryoo@snu.ac.kr & carpediemwj@snu.ac.kr

**Supplementary Figure 1. RNA-seq analysis on the DEGs regulated by NAM treatment for 4 or 10 days in MC3T3-E1 cells. Related to Figure 1.**

(a-b) The differential expression of genes by NAM were visualized using MA plots.

(c) GO analysis was performed using upregulated and downregulated DEGs by NAM on day 4 and 10. GO terms were selected and sorted based on enrichment score.

**h**

**Supplementary Figure 2. Correlation analysis of DEGs upregulated by NAM on day 4. Related to Figure 1.**

(a) Correlation analysis of upregulated genes by NAM on day 4. A normalized correlation matrix was performed to show correlation among GO terms.

(b) GO analysis was performed with genes included in each cluster. GO terms commonly included in all clusters are indicated.

(c-g) GO analysis of genes enriched in each cluster are displayed.

(h) GO enrichment analysis results of cluster 4 on day 4.

**j**

**Supplementary Figure 3. Correlation analysis of DEGs upregulated by NAM on day 10. Related to Figure 2.**

(a) Correlation analysis of upregulated genes by NAM on day 10. A normalized correlation matrix was performed to show correlation among GO terms.

(b) GO analysis was performed with genes included in each cluster. GO terms commonly included in all clusters are indicated.

(c-i) GO analysis of genes enriched in each cluster are displayed.

(j) GO enrichment analysis results of cluster 4 on day 10.

**Supplementary Figure 4. The measurement of mitochondrial respiration using XF96 Extracellular Flux Analyzer. Related to Figure 4.**

(a-h) MC3T3-E1 cells were cultivated in osteogenic medium for indicated days and the Oxygen Consumption Rate (OCR) was measured with an XF96 Extracellular Flux Analyzer (n≥3). The summary of curved OCR plot (a) was calculated and displayed with bar plots (b-h).

(i-l) MC3T3-E1 cells were cultured with indicated concentrations of NAM in growth medium for 1d and OCR was measured. (m-p) MC3T3-E1 cells were cultured with 10 μM of NAM in osteogenic medium for 7 days.

**Supplementary Figure 5. RNA-seq analysis of H_2_O_2_-induced gene expression in osteoblasts. Related to Figure 5.**

(a) Cells were cultured with NAM (10 μM) and H_2_O_2_ (100 μM) in the osteogenic medium including β-glycerophosphate and ascorbic acid. The medium containing NAM and H_2_O_2_ were changed freshly every two days. Day 0 defines the start of differentiation with NAM and H_2_O_2_.

(b-c) GO analysis of upregulated or downregulated DEGs by 100 μM of H_2_O_2_ on day 4. The top 20 GO terms are selected based on adjusted p-value and sorted by enrichment score.

(d-e) GO analysis of upregulated or downregulated DEGs by 100 μM of H_2_O_2_ on day 10. The top 20 GO terms are selected based on adjusted p-value and sorted by enrichment score.

**a**

**b c**


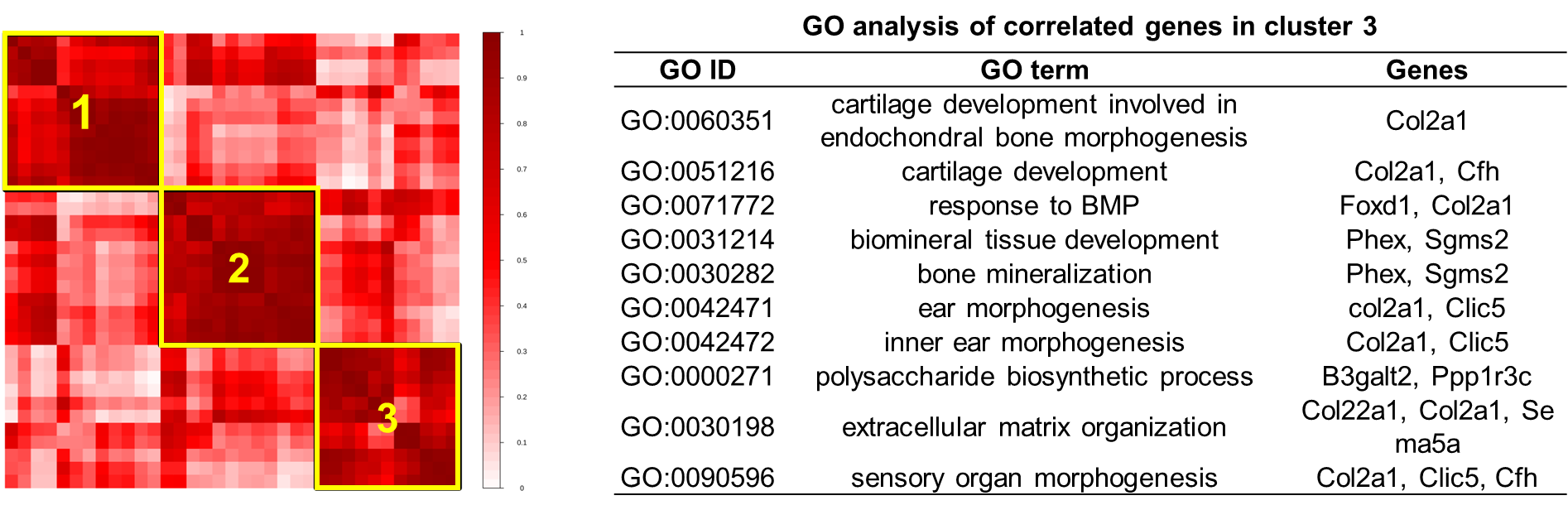


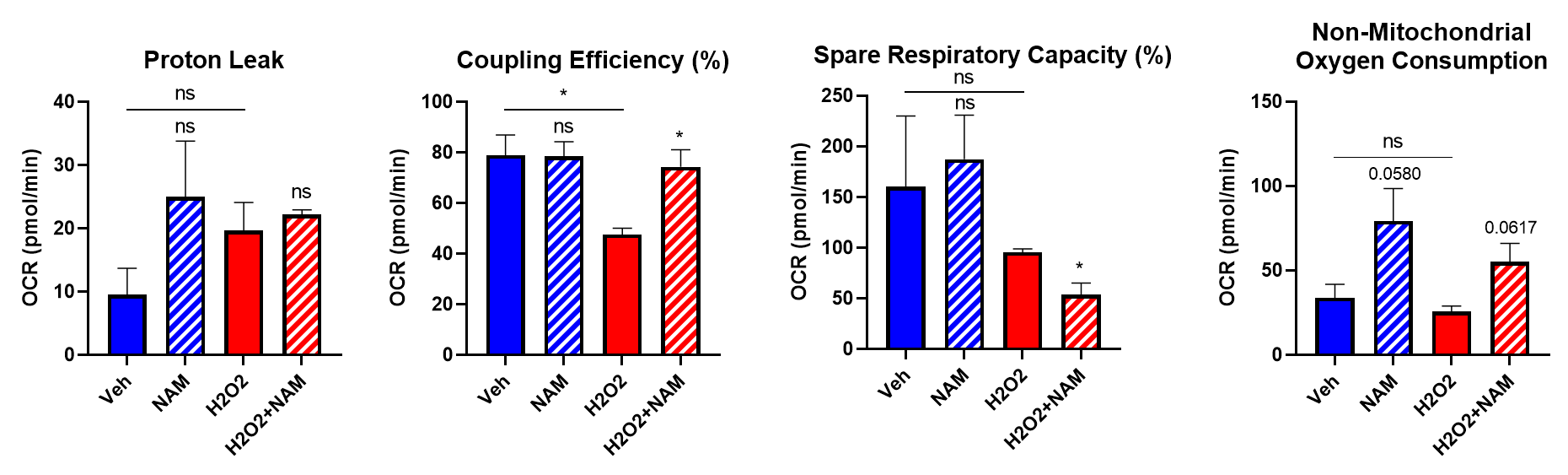
**d e f g**

**Supplementary Figure 6. NAM prevents ROS-induced damage in osteoblasts. Related to Figure 5.**

(a) H_2_O_2_-decreased DEGs restored by NAM on day 4 are listed in table.

(b) Correlation matrix plot on the DEGs whose expression was decreased by H_2_O_2_-restored by NAM, included in top 20 GO category.

(c) The list of genes included in cluster 3 of correlation analysis (b).

(d-g) The mitochondrial OCR was evaluated after the treatment of 10 μM NAM together with or without 100 μM H_2_O_2_ for 7 d in osteogenic media using an XF96 Extracellular Flux Analyzer. The parameters calculated from the curved OCR plot (Figure 5e) were described into bar plots.

**Supplementary Table 1. Primer sequences for RT-qPCR**

| **gene** | **sequence** |
| --- | --- |
| ***Runx2*** | F: 5’-CCGCACGACAACCGCACCAT-3’ |
|  | R: 5’-CGCTCCGGCCCACAAATCTC-3’ |
| ***Osx*** | F: 5’-CCCACCCTTCCCTCACTCAT-3’ |
|  | R: CCT TGT ACC ACG AGC CAT AGG-3’ |
| ***Dlx5*** | F: 5’-TCTCTAGGACTGACGCAAACA-3’ |
|  | R: GTTACACGCCATAGGGTCGC-3’ |
| ***Bsp*** | F: 5’-CAG GGA GGC AGT GAC TCT TC-3’ |
|  | R: AGT GTG GAA AGT GTG GCG TT-3’ |
| ***Ocn*** | F: 5’-CTG ACA AAG CCT TCA TGT CCA A -3’ |
|  | R: GCG CCG GAG TCT GTT CAC TA -3’ |
| ***Opn*** | F: 5’-ATC TCA CCA TTC GGA TGA GTC T-3’ |
|  | R: TCA GTC CAT AAG CCA AGC TAT CA-3’ |
| ***Mepe*** | F: 5’-GTCTGTTGGACTGCTCCTCTT-3’ |
|  | R: CACCGTGGGATCAGGATACA-3’ |
| ***Pgc1-α*** | F: 5’-ACTATGAATCAAGCCACTACAGAC-3’ |
|  | R: TTCATCCCTCTTGAGCCTTTCG-3’ |
| ***Ucp2*** | F: 5’-TAAAGGTCCGCTTCCAGGCTCA-3’ |
|  | R: ACGGGCAACATTGGGAGAAGTC-3’ |
| ***Trx2*** | F: 5’-CCTTTAACGTCCAGGATGGACC-3’ |
|  | R: TTGGCGACCATCTTCTCTAGCC-3’ |
| ***Sod1*** | F: 5’-GGTGAACCAGTTGTGTTGTCAGG-3’ |
|  | R: ATGAGGTCCTGCACTGGTACAG-3’ |
| ***Sod2*** | F: 5’-TAACGCGCAGATCATGCAGCTG-3’ |
|  | R: AGGCTGAAGAGCGACCTGAGTT-3’ |
| ***Sirt3*** | F: 5’-GCTACATGCACGGTCTGTCGAA-3’ |
|  | R: CAATGTCGGGTTTCACAACGCC-3’ |
| ***Gapdh*** | F: 5’-CATGTTCCAGTATGACTCCACTC-3’ |
|  | R: 5’-GGCCTCACCCCATTTGATGT-3’ |

**Supplementary Table 2. GO analysis of correlated genes in cluster 1**

| **GO ID** | **GO term** | **Genes** |
| --- | --- | --- |
| GO:0071772 | response to BMP | Hipk2, Smpd3, Runx2 |
| GO:0031214 | biomineral tissue development | Co1a2, Smpd3, Wnt10b |
| GO:0030282 | bone mineralization | Col1a2, Smpd3, Wnt10b |
| GO:0042476 | odontogenesis | Smpd3, Pdgfra, Runx2 |
| GO:0042475 | odontogenesis of dentin-containing tooth | Smpd3, Pdgfra, Runx2 |
| GO:0048562 | embryonic organ morphogenesis | Chd7, Hipk2, Pdgfra, Runx2 |
| GO:0030198 | extracellular matrix organization | Loxl4, Col1a2, Smpd3, Pdgfra |
| GO:0048705 | skeletal system morphogenesis | Smpd3, Pdgfra, Runx2 |
| GO:0060541 | respiratory system development | Chd7, Smpd3, Pdgfra, Klf2 |
| GO:0034614 | cellular response to reactive oxygen species | Hk3, Map3k5, Smpd3, Pdgfra |

**Supplementary Table 3. GO analysis of correlated genes in cluster 2**

| **GO ID** | **GO term** | **Genes** |
| --- | --- | --- |
| GO:0060351 | cartilage development involved in endochodral bone morphogenesis | Mmp13 |
| GO:0051216 | cartilage development | Mmp13, Bmp1 |
| GO:0042471 | ear morphogenesis | Tmie, Dlx5 |
| GO:0042472 | inner ear morphogenesis | Tmie, Dlx5 |
| GO:0060350 | endochondral bone morphogenesis | Mmp13, Dlx5 |
| GO:0090596 | sensory organ morphogenesis | Tmie, Fzd5, Dlx5 |
| GO:0048705 | skeletal system morphogenesis | Mmp13, Dhrs3, Dlx5 |
| GO:0060348 | bone development | Mmp13, Dhrs3, Dlx5 |
| GO:0061138 | morphogenesis of a branching epithelium | Vdr, Tnc, Fzd5 |
| GO:0060349 | bone morphogenesis | Mmp13, Dhrs3, Dlx5 |
